# Associations of creatinine/cystatin C ratio and postoperative pulmonary complications in elderly patients undergoing off-pump coronary artery bypass surgery: a retrospective study

**DOI:** 10.1038/s41598-021-96442-0

**Published:** 2021-08-19

**Authors:** Hye Jin Kim, Hye-Bin Kim, Ha Yan Kim, Jae-Kwang Shim, Cheolhun Lee, Young-Lan Kwak

**Affiliations:** 1 Department of Anaesthesiology and Pain Medicine, Anaesthesia and Pain Research Institute, Yonsei University College of Medicine, Seoul, 03722 South Korea; 2 Biostatistics Collaboration Unit, Department of Biomedical Systems Informatics, Yonsei University College of Medicine, Seoul, 03722 South Korea

**Keywords:** Risk factors, Outcomes research

## Abstract

Sarcopenia along with nutritional status are associated with postoperative pulmonary complications in various surgical fields. Recently, the creatinine/cystatin C ratio and CONtrolling NUTritional status score were introduced as biochemical indicators for sarcopenia and malnutrition, respectively. We aimed to investigate the associations among these indicators and postoperative pulmonary complications in elderly patients undergoing off-pump coronary artery bypass surgery. We reviewed the medical records of 605 elderly patients (aged ≥ 65 years) who underwent off-pump coronary artery bypass surgery from January 2010 to December 2019. Postoperative pulmonary complications (pneumonia, prolonged ventilation [> 24 h], and reintubation during post-surgical hospitalisation) occurred in 80 patients. A 10-unit increase of creatinine/cystatin C ratio was associated with a reduced risk of postoperative pulmonary complications (odds ratio: 0.80, 95% confidence interval: 0.69–0.92, *P* = 0.001); the optimal cut-off values for predicting postoperative pulmonary complications was 89.5. Multivariable logistic regression analysis revealed that age, congestive heart failure, and creatinine/cystatin C ratio < 89.5 (odds ratio 2.36, 95% confidence interval 1.28–4.37) were independently associated with the occurrence of postoperative pulmonary complications, whereas CONtrolling NUTritional status score was not. A low creatinine/cystatin C ratio was associated with an increased risk of developing postoperative pulmonary complications after off-pump coronary artery bypass surgery.

## Introduction

Confronting the challenges of an aging society, the prognostic importance of sarcopenia along with malnutrition has been highlighted as core concept of frailty because they are potentially modifiable factors that may improve outcomes in older adult patients requiring surgery^1^. Sarcopenia, which manifests as low muscle strength with low muscle quantity or quality, is observed in 25–60% of patients undergoing major surgeries^1–3^. While computed tomography (CT) and magnetic resonance imaging (MRI) are the gold standard techniques for the measurement of muscle quantity^4^, they may not be readily feasible for general use in terms of accessibility and socioeconomic efficiency^5^. In this context, the creatinine/cystatin C (CysC) ratio, a recently introduced objective biochemical sarcopenia indicator^5^, may be considered as a practical alternative.

Both serum creatinine and CysC levels reflect glomerular filtration rate based on muscle and nucleated cells, respectively^6^, indicating that the creatinine/CysC ratio potentially reflects muscle mass. The creatinine/CysC ratio has shown a strong association with CT-assessed muscle quantity in patients with cancer (no disclosure of the specific correlation method; Pearson’s or Spearman’s r = 0.648, *P* < 0.001), critically ill status (Pearson’s r = 0.62, *P* < 0.001), and lung transplant surgery (Pearson’s r = 0.43, *P* = 0.02)^5,7,8^. Additionally, a statistically significant correlation with muscle strength derived from a handgrip test (Spearman’s r = 0.54, *P* < 0.01) and a good performance on discriminating sarcopenia (AUROC = 0.87) in patients with chronic obstructive pulmonary disease have been reported^9^.

Among the complications related to sarcopenia, postoperative pulmonary complications (PPCs) are common after cardiac surgery; this complication is often associated with detrimental prognosis^10,11^. Despite a seemingly beneficial effect on the lungs by avoiding cardiopulmonary bypass, off-pump coronary artery bypass surgery (OPCAB) continues to be associated with a high prevalence of PPCs, ranging from 3.1 to 11.4%^12–14^. Thus, the identification of objective and modifiable preoperative risk factors, such as sarcopenia, for PPCs in older adult patients undergoing OPCAB would be of clinical significance to improve postoperative recovery through early rehabilitation. In addition, malnutrition, a factor closely related to sarcopenia, has been defined by an objective biochemical nutritional index, CONtrolling NUTritional status (CONUT) score^15^. It, too, has been associated with PPCs and survival in patients undergoing cancer resection^16,17^. The CONUT scoring includes an immune indicator (total lymphocyte count), a caloric depletion parameter (cholesterol), and traditional serum protein markers (albumin concentration). In addition to the nutritional status, CONUT score also reflects the patient’s immunologic status^15^, both of which could be closely interlinked with PPCs. Despite the interrelationship between sarcopenia, malnutrition, and PPCs, limited evidence is available on the relevance of the objective indices (creatinine/CysC ratio and CONUT score, respectively) and occurrence of PPCs in older patients undergoing OPCAB.

In this retrospective, single-centre study, we investigated the associations between the creatinine/CysC ratio and CONUT score and PPCs (primary outcomes) and other morbidities/mortality outcomes (secondary outcomes) in older patients undergoing OPCAB.

## Methods

### Patients

The institutional review board of Severance Hospital, Seoul, South Korea (IRB no. 4-2020-1128 on November 26, 2020) approved this study and waived the requirement for written informed consent. This study was registered at ClinicalTrials.gov (NCT04654663; first posted: December 4, 2020) and conducted according to the 2013 Declaration of Helsinki. This manuscript adheres to the Strengthening the Reporting of Observational Studies in Epidemiology guidelines. Data were retrospectively collected from electronic medical records of patients who underwent OPCAB at the Severance Cardiovascular Hospital, Yonsei University Health System, Seoul, Korea, from January 2010 to December 2019.

Patients aged ≥ 65 years with serum CysC and creatinine test results obtained within 1 week before surgery were enrolled. Patients with chronic kidney disease (estimated glomerular filtration rate < 60 mL min^−1^ 1.73 m^−2^), acute kidney injury, or respiratory tract infection requiring medical therapy that occurred within 1 month before surgery were excluded. Definition of acute kidney injury was based on the Kidney Disease: Improving Global Outcomes criteria^18^.

### Data collection

All operations were performed by two expert surgeons (Y.K.J. and Y.Y.N.) with an experience of > 100 surgeries. All data were extracted from the patients’ electronic medical charts and prospectively recorded protocol forms. The baseline patient demographic data included age, sex, height, weight, Body mass index (BMI), nature of the operation (emergency operation and/or reoperation), EuroSCORE II, smoking status, and history of hypertension, diabetes mellitus, cerebrovascular disease, congestive heart failure, chronic obstructive pulmonary disease, and myocardial infarction within 1 month before surgery. Preoperative serum creatinine, CysC, albumin, cholesterol, C-reactive protein, and blood haemoglobin levels and lymphocyte count were also recorded. The CONUT score was calculated based on Ulíbarri’s study^15^. A CONUT score of < 5 (normal or light undernutrition) was regarded as a low CONUT grade, whereas a CONUT score of ≥ 5 (moderate-to-severe undernutrition) was regarded as a high CONUT grade. Anaemia was defined as a haemoglobin level < 12.0 g/dL for women and < 13.0 g/dL for men, according to the World Health Organization guidelines. The estimated glomerular filtration rate was calculated from patient serum creatinine levels using the chronic kidney disease epidemiology collaboration equation^19^. Additionally, the presence of significant (> 50%) left main coronary stenosis on coronary angiography and moderate or higher mitral regurgitation grades on preoperative echocardiography and use of preoperatively administered medications (aspirin, clopidogrel, beta-blockers, calcium channel blockers, renin-angiotensin system antagonists, insulin, and 3-hydroxy-3-methylglutaryl coenzyme A reductase inhibitors) were recorded.

The intraoperative and postoperative data (for 24 h after the operation), including the number of grafts anastomosed, duration of operation/anaesthesia, fluid and transfusion requirement, amount of cell saver infusion, and urine output and chest tube drainage, were assessed. Intensive care unit (ICU) and hospital stay length and major post-surgical complications (pneumonia, prolonged ventilation [> 24 h], reintubation, renal failure, delirium, reoperation due to bleeding/tamponade, permanent stroke, and mortality) were also evaluated. All complications, except pneumonia, were defined based on the STS Adult Cardiac Surgery Database Data Specifications version 4.20.2^20^; pneumonia followed the definition of the European Perioperative Clinical Outcome^21^. All complications, except for mortality, were limited to the occurrence of an event during hospitalisation for surgery. Mortality included events occurring after hospital discharge through the end of the thirtieth postoperative day^20^.

### Study endpoint

The primary endpoints were the association of the creatinine/CysC ratio and CONUT score with PPCs (pneumonia, prolonged ventilation [> 24 h], and reintubation).

The secondary endpoints were the association of the indices with morbidity endpoints (renal failure, delirium, reoperation due to bleeding/tamponade, permanent stroke, and mortality).

### Statistical analyses

Patients were divided into two groups based on the occurrence of PPCs. To make intergroup comparisons, the continuous variables were analysed using an independent t-test or Mann–Whitney U test based on the results of a Shapiro–Wilk normality test. Categorical variables were analysed using a Chi-squared test or Fisher’s exact test. Binary data were presented as numbers (%), whereas continuous data were presented as either a mean ± standard deviation or median (interquartile range) based on the distribution.

A binary logistic regression analysis was performed, with or without adjusting for the EuroSCORE II, to determine the association between the creatinine/CysC ratio and morbidity endpoints. The area under the receiver operating characteristic curve (AUROC) was also calculated. By maximising the Youden index, the optimal cut-off value was determined and utilised for classifying the high and low creatinine/CysC ratio groups.

Age, sex, potential risk factors (*P* value < 0.05 in the univariable analysis), CONUT grade, and the creatinine/CysC ratio (either continuous or binary variables with a cut-off value of 89.5) were included in the multivariable binary logistic model for the occurrence of PPCs. The rule of ten was applied to each model to avoid overfitting, and the model with the highest concordance statistics was selected. The statistical significance was set at a *P* value of < 0.05.

All analyses were performed using SPSS version 25 software (IBM Corp., Armonk, NY, USA), R version 4.0.2 (The R Foundation for Statistical Computing, Vienna, Austria), and SAS (version 9.4, SAS Inc., Cary, NC, USA).

## Results

**Figure 1:**
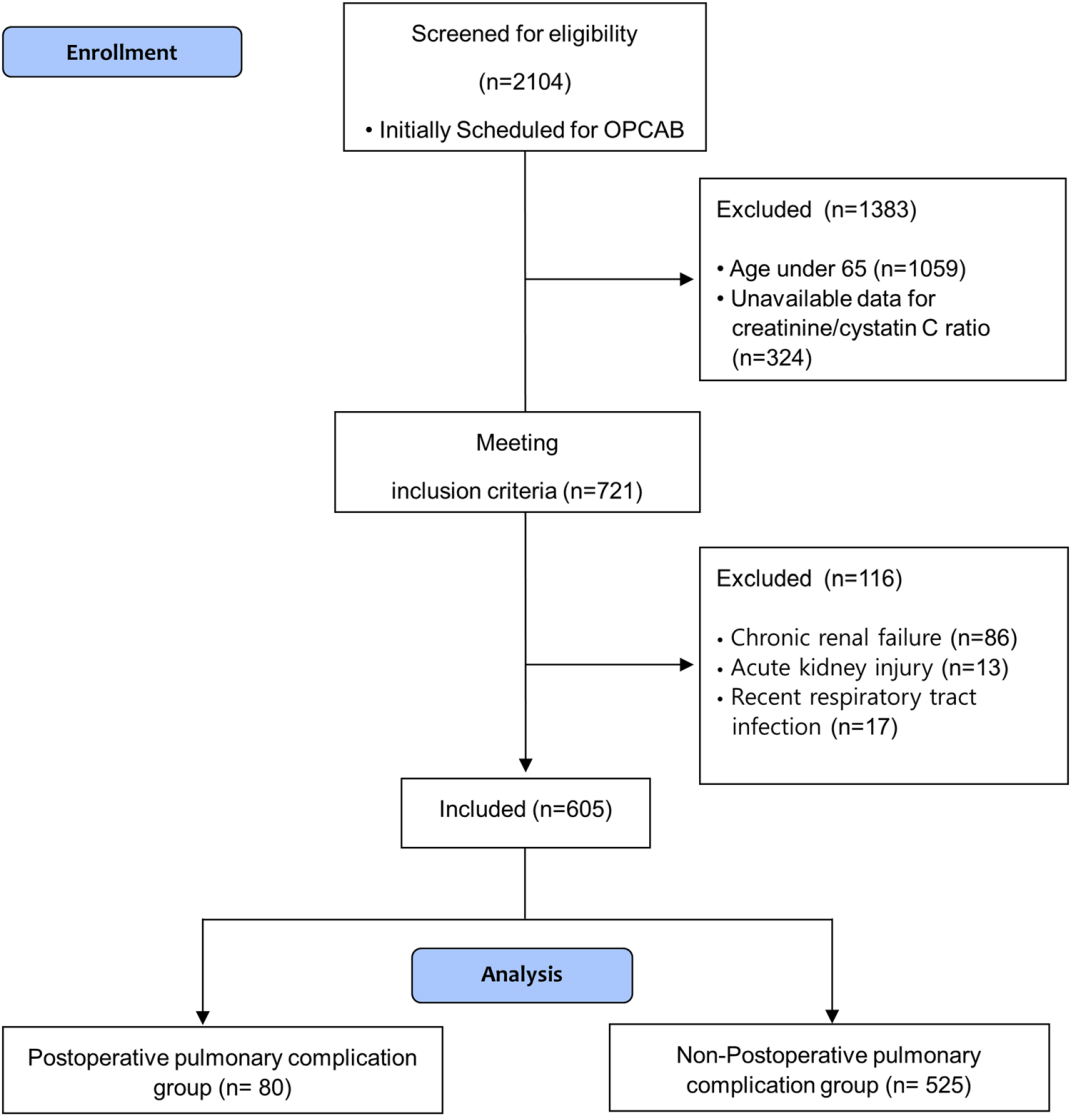
Flow chart of patient enrolment. Among the 2104 patients scheduled for off-pump coronary artery bypass graft surgery from January 2010 to December 2019, 1383 did not meet the inclusion criteria (1059 patients were < 65 years old, and 324 did not have serum cystatin C and creatinine test results that were obtained within 1 week before surgery). Among the remaining 721 patients, 116 met the exclusion criteria (86 patients had chronic renal failure, 13 had acute kidney injuries, and 17 had respiratory tract infections within 1 month before surgery). Finally, 605 patients were enrolled and divided into two groups according to the occurrence of postoperative pulmonary complications.

We initially screened 2104 patients who underwent OPCAB between 2010 and 2019. Among them, 1383 patients were excluded, and finally, 605 patients were enrolled (Fig. 1). No data regarding the outcome endpoints were missing. In other variables, except for intraoperative blood loss (189 missing data) and the amount of cell saver infusion (18 missing data), there were none or less than 10 data missing occurred. Missing data were excluded from analyses.

A total of 80 (13.2%) patients experienced PPCs. Patients in the PPC group showed a statistically significant lower creatinine/CysC ratio than did those in the non-PPC group (80.6 ± 16.5 vs 87.5 ± 18.2, *P* = 0.001). Patients in the PPC group were older with lower BMI, higher EuroSCORE II, and CONUT score and were more likely to undergo emergent OPCAB than patients in the non-PPC group were. In the PPC group, patients were more frequently diagnosed with anaemia and congestive heart failure than those in the non-PPC group were (Table 1). Preoperative laboratory data demonstrated a lower serum albumin concentration in the PPC group (3.8 ± 0.5 g dL^−1^ vs. 4.0 ± 0.4 g dL^−1^, *P* = 0.001) than in the non-PPC group (Supplementary Table S1).

**Table 1:** Baseline patient characteristics.

Variables	PPC group (n = 80)	Non-PPC group (n = 525)	*P *value
Age (years)	73.4 ± 4.3	71.5 ± 4.4	< 0.001
Male sex	55 (68.8)	384 (73.1)	0.412
Emergency	5 (6.3)	10 (1.9)	0.037
BMI (kg/m^2^)	23.5 [20.7–25.1]	24.0 [22.3–25.9]	0.004
EuroSCORE II	1.36 [1.13–1.84]	1.18 [0.94–1.57]	0.001
Current smoker	11 (13.8)	68 (13.0)	0.844
Hypertension	64 (80.0)	395 (75.2)	0.354
Diabetes mellitus	39 (48.8)	264 (50.3)	0.798
CVA	13 (16.3)	67 (12.8)	0.391
CHF	17 (21.3)	42 (8.0)	< 0.001
COPD	5 (6.3)	25 (4.8)	0.578
MI within 1 month	29 (36.3)	153 (29.1)	0.197
Left main > 50% stenosis	23 (28.7)	152 (29.0)	0.970
Mitral regurgitation ≥ moderate	7 (8.8)	25 (4.8)	0.174
**Preoperative medications**			
Aspirin	69 (86.3)	451 (85.9)	0.934
Clopidogrel	50 (62.5)	299 (57.0)	0.349
Beta-blocker	39 (48.8)	297 (56.6)	0.190
Calcium channel blocker	36 (45.0)	221 (42.1)	0.624
RAS inhibitor	44 (55.0)	300 (57.1)	0.718
Insulin	5 (6.3)	46 (8.8)	0.451
HMG-CoA reductase inhibitors	63 (78.8)	420 (80.0)	0.795
Reoperation	1 (1.3)	4 (0.8)	0.509
Anaemia	45 (56.3)	223 (42.5)	0.021
CONUT	2 (0–8)	2 (0–11)	0.020
Creatinine/cystatin C ratio	80.6 ± 16.5	87.5 ± 18.2	0.001

Values are presented as mean ± standard deviation, median [interquartile range], or the number of patients (percent). The CONtrolling NUTritional status score is expressed as median (range). BMI, body mass index; PPC, postoperative pulmonary complication; RAS, renin-angiotensin system; CHF, congestive heart failure; CVA, cerebrovascular accident; COPD, chronic obstructive pulmonary disease; MI, myocardial infarction; CONUT, CONtrolling NUTritional status.

Intra- and post-operative data, including fluid balance, were comparable between the PPC and non-PPC groups (Table 2).

**Table 2:** Intra- and post-operative data.

Variables	PPC group (n = 80)	Non-PPC group (n = 525)	*P *value
**Intraoperative data**			
Graft number	3 [3–4]	3 [3–4]	0.816
Duration of anaesthesia (mins)	298 [270–333]	300 [275–325]	0.761
Duration of operation (mins)	225 [200–252]	228 [205–250]	0.720
Total fluid input (mL)	2443 ± 753	2318 ± 845	0.215
Urine output (mL)	240 [135–403]	280 [150–475]	0.097
Blood loss (mL)	530 [370–740]	610 [480–790]	0.074
Cell saver infusion (mL)	219 [130–245]	220 [170–270]	0.139
Number of patients who had pRBC transfused	23 (28.7)	112 (21.3)	0.138
Number of patients who had blood transfused	24 (30.0)	129 (24.6)	0.298
**Postoperative data for 24 h**			
Total fluid input (mL)	3730 [3155–4210]	3736 [3269–4290]	0.722
Urine output (mL)	2550 [2035–3090]	2670 [2085–3200]	0.404
Chest tube drainage (mL)	720 [495–880]	700 [530–870]	0.796
Number of patients who had pRBC transfused	26 (32.5)	144 (27.4)	0.347
Number of patients who had blood transfused	35 (43.8)	204 (38.9)	0.404

Values are presented as means ± standard deviations, medians [interquartile ranges], or the number of patients (percentages). PPC, postoperative pulmonary complication; pRBC, packed red blood cell.

The odds ratio of creatinine/CysC ratio per 10 units for the occurrence of postoperative morbidity endpoints is presented in Table 3. A 10-unit increase of creatinine/CysC ratio (continuous variable) was associated with a reduced risk of PPC occurrence with (odds ratio: 0.82, 95% confidence interval [CI]: 0.72–0.95, *P* = 0.010) or without adjustments (odds ratio: 0.80, 95% CI: 0.69–0.92, *P* = 0.001) for EuroSCORE II. Additionally, permanent stroke was negatively associated with creatinine/CysC ratio) (per 10 units) after adjustments for EuroSCORE II (odds ratio: 0.45, 95% CI: 0.21–0.95, *P* = 0.036). An increase of one unit of creatinine/CysC ratio was associated with decreased ICU and hospital stay lengths (regression coefficient: − 0.035, 95% CI: − 0.0526 to − 0.0174, *P* = 0.0002 and regression coefficient: − 0.122, 95% CI: − 0.1690 to − 0.0770, *P* < 0.0001, respectively).

**Table 3:** Odds ratio for the creatinine/cystatin C ratio per 10 units (continuous variable) for the occurrence of postoperative complications.

Outcomes	Unadjusted OR (95% CI)	*P* value	^a^Adjusted OR (95% CI)	*P* value
**Pulmonary complications**	0.80 (0.69–0.92)	0.001	0.82 (0.72–0.95)	0.010
Pneumonia	0.74 (0.59–0.92)	0.008	0.75 (0.60–0.94)	0.013
Prolonged ventilation	0.83 (0.72–0.97)	0.021	0.87 (0.74–1.02)	0.087
Reintubation	0.73 (0.56–0.93)	0.012	0.75 (0.58–0.97)	0.030
**Renal failure**	0.74 (0.55–1.01)	0.057	0.75 (0.55–1.03)	0.072
**Delirium**	0.89 (0.79–1.00)	0.057	0.92 (0.81–1.03)	0.144
**Reoperation due to bleeding/tamponade**	1.11 (0.82–1.49)	0.501	1.10 (0.81–1.49)	0.539
**Permanent stroke**	0.49 (0.24–1.03)	0.061	0.45 (0.21–0.95)	0.036
**Mortality**	0.79 (0.59–1.06)	0.109	0.81 (0.61–1.09)	0.173

^a^Adjusted by EuroSCORE II.

OR, odds ratio; CI, confidence interval.

The optimal cut-off values for predicting PPCs were 89.5 (sensitivity, 77.5%; specificity, 45.5%; AUROC, 0.615) for both sexes—90.5 (sensitivity, 76.4%; specificity, 56.8%) for males and 71.7 (sensitivity, 64.0%; specificity, 51.8%) for females. Patients with a low creatinine/CysC ratio were older and more frequently women than patients with a high creatinine/CysC ratio were; they also had a greater incidence of emergent operation, congestive heart failure, moderate or higher mitral regurgitation, and lower haemoglobin, lower albumin, and higher C-reactive protein levels than patients with a high creatinine/CysC ratio did (Supplementary Table S2). A low creatinine/CysC ratio was associated with not only an increased risk of PPC occurrence but also an increased risk of renal failure and mortality. This association continued even after adjustment for EurSCORE II (Supplementary Table S3).

Patients with a high CONUT grade had a lower BMI, higher EuroSCORE II, greater incidence of cerebrovascular accident, myocardial infarction within within 1 month, and anaemia, lower creatinine/CysC ratio, and higher C-reactive protein levels than those with a low CONUT grade did (Supplementary Table S4). A high CONUT grade was associated with an increased risk of PPC and delirium (Supplementary Table S5).

In the multivariable model for identifying risk factors for PPCs, low creatinine/CysC ratio was found to be an independent risk factor (odds ratio: 2.36, 95% CI: 1.28–4.34, *P* = 0.006), whereas an increase in ten units of creatinine/CysC ratio was not independently associated with the occurrence of PPCs (odds ratio: 0.84, 95% CI: 0.71–1.01, *P* = 0.060). Unlike the CONUT score ≥ 5, age and congestive heart failure were independently associated with the occurrence of PPCs (Table 4).

**Table 4:** Univariable and multivariable analysis for postoperative pulmonary complications.

Variables	Univariable		Variable	Multivariable	
	OR (95% CI)	*P* value		OR (95% CI)	*P* value
Age (years)	1.10 (1.05–1.16)	< 0.001	Age (years)	1.07 (1.01–1.13)	0.016
Male sex	1.24 (0.74–2.06)	0.413	Male sex	0.88 (0.49–1.56)	0.655
BMI (kg/m^2^)	0.89 (0.82–0.97)	0.005	BMI (kg/m^2^)	0.93 (0.85–1.01)	0.078
Current smoker	1.07 (0.54–2.13)	0.844			
Emergency	3.43 (1.14–10.32)	0.028	Emergency	2.45 (0.77–7.76)	0.128
CHF	3.10 (1.67–5.78)	< 0.001	CHF	2.62 (1.35–5.06)	0.004
COPD	1.33 (0.50–3.59)	0.569			
Reoperation	1.65 (0.18–14.94)	0.657			
Anaemia	1.74 (1.08–7.80)	0.022	Anaemia	1.43 (0.85–2.39)	0.176
CONUT score ≥ 5	2.59 (1.28–5.26)	0.008	CONUT score ≥ 5	1.67 (0.77–3.66)	0.196
Creatinine/cystatin C ratio < 89.5	2.88 (1.66–5.00)	< 0.001	Creatinine/cystatin C ratio < 89.5	2.36 (1.28–4.34)	0.006
Creatinine/cystatin C ratio (per 10 units)	0.80 (0.69–0.92)	0.001	Concordance statistics	0.720	

OR, odds ratio; CI, confidence interval; BMI, body mass index; CONUT, CONtrolling NUTritional status; CHF, congestive heart failure; COPD, chronic obstructive pulmonary disease.

## Discussion

In our retrospective study, a creatinine/CysC ratio of < 89.5, a surrogate marker for sarcopenia, was independently associated with occurrence of PPCs in older adult patients who underwent OPCAB along with age and congestive heart failure, whereas the CONUT score was not. Additionally, a low creatinine/CysC ratio was related to an increased risk of permanent stroke, renal failure, mortality, and prolonged ICU/hospital stays after surgery.

The prevalence of PPCs after cardiac surgery is 27–35%^10,22,23^. In multivessel OPCAB, median sternotomy, internal mammary artery dissection, and lung manipulation are still necessary, resulting in direct lung injury and severe pain^10^. Thus, despite avoiding cardiopulmonary bypass, PPC remains one of the leading complications after OPCAB, which further progresses to other morbidities or death^10^. If a correctible vulnerable target is detected in advance, followed by preventive or patients-specific care, it is expected to enhance recovery and reduce socioeconomic costs^24^. Preoperative sarcopenia has been associated with PPCs in cancer cohorts^1,25^. Sarcopenia is closely related to dysphagia and respiratory muscle weakness^26^, and it is observed at a high frequency in patients with respiratory failure^27^. Likewise, an increased incidence of community-acquired pneumonia was reported in older patients with sarcopenia^28^. Moreover, low muscle mass may imply impaired immunity or inflammation^3^, which also enhances the risk of PPCs.

The recently proposed creatinine/CysC ratio is easily obtained with a simple calculation using serum creatinine and CysC levels^5^. Since serum creatinine originates from muscle catabolism and CysC is produced by nucleated cells^6^, a low creatinine/CysC ratio implies low muscle mass and, thus, sarcopenia. In our study, low creatinine/CysC ratio, as a binary variable (< 89.5) implying sarcopenia, was independently associated with PPCs after OPCAB, while creatinine/CysC ratio, as a continuous variable, only showed an association with PPCs in the univariable analysis. Binary instead of a linear value appeared to be a more meaningful parameter for the occurrence of PPC. In other words, knowing whether the muscle is normal or sarcopenic is more important than knowing the absolute value of the muscle mass; if the muscle mass is within the normal range, an increase in it might not reduce the risk of PPCs. In agreement with our results, previous studies using CT for diagnosing sarcopenia that implemented a binary approach showed an association between sarcopenia and pulmonary morbidity in critically ill patients^29,30^ and surgical patients with colon and oesophageal cancers^1,31^. Some of these studies examined the linear relationship between muscle mass and pulmonary morbidity; however, the results were inconsistent^29,30^, showing a weak linear relationship.

Additionally, in our study, low creatinine/CysC ratio was associated with not only PPCs but also with other morbidity endpoints, such as permanent stroke, renal failure, mortality, and longer ICU and hospital stay lengths, implicating a more generalised effect in terms of outcome. Similarly, a correlation has been reported between low creatinine/CysC ratio and poor prognosis in critically ill patients^5,32,33^. In a retrospective study of 226 critically ill patients (stable kidney disease), a subgroup analysis involving patients on invasive mechanical ventilators (n = 131) revealed an association between higher creatinine/CysC ratio and shorter duration of mechanical ventilation^5^. In another retrospective study involving neurocritically ill patients (n = 538, with stable kidney function), the creatinine/CysC ratio was significantly correlated with the duration of mechanical ventilation, risk of tracheostomy, and length of neurocritical care unit^33^. A recent study in older adult patients who underwent percutaneous coronary intervention demonstrated that a low creatinine/CysC ratio increased the risk of major adverse cardiovascular events and mortality^34^. As a primary site of glucose uptake and protein reservoir, skeletal muscle supplies fuel and ingredients for the body to function and to recover^35^. Thus, sarcopenia indicates a lack of reserve for coping with external stress; it could be related to poor prognosis in critically ill and surgical patients^3–5^.

In contrast to our expectation, the CONUT score was not independently associated with PPC in our study. In terms of causality, sarcopenia takes a long time to develop. It is affected not only by malnutrition but also by various factors, such as physical inactivity and comorbidities^36^. In a study on patients with respiratory failure requiring mechanical ventilation^27^, a significant number of patients classified as normally nourished were found to be sarcopenic. Muscle is an important protein storehouse during fasting or stressful conditions^37^. Surgery intensifies a patient’s catabolic status, increasing protein demand beyond the amount supplied by the diet^38^; therefore, the muscle mass is more clinically relevant as a nutritional reserve during the perioperative period. Our results imply that a low creatinine/CysC ratio is more appropriate than the CONUT score for selecting OPCAB patients vulnerable to PPCs. For these patients, preoperative physiotherapy, and protein supplementation to correct sarcopenia might be effective interventions for preventing the occurrence of PPCs^35,39,40^, although verification through further research is needed.

Our study has several strengths. Since the creatinine/CysC ratio, an easily acquired and objective indicator, is not influenced by the operator, it can be utilised as a screening test. Moreover, it can be applied to various patient groups (neurocognitive impaired patients, bed-ridden patients, and patients with unstable vital signs) and in various clinical situations (emergent operation and critical care unit), as it does not require patient effort or cooperation or any shift in the position. We are the first to analyse the effect of creatinine/CysC ratio on the occurrence of PPCs in patients undergoing OPCAB. Despite the disadvantage of being a retrospective study, there were no missing data related to the primary outcomes, and several possible confounding factors were controlled in the analysis.

However, our study had some limitations. First, in addition to the inherent limitations of a retrospective study, muscle quality or strength was not measured in our study. Although creatinine/CysC ratio has been previously shown to correlate with the handgrip test^9^, it reflects muscle mass rather than muscle strength or quality. Whether the combined use of creatinine/CysC ratio and handgrip test will improve the predictability of PPC occurrence in cardiac surgery patients needs further investigation. Second, the types of patients eligible for inclusion were limited. Given that chronic kidney disease critically affects fluid balance and is an important risk factor for PPCs^21,41^, we tried to exclude its effect by limiting enrolments of such patients and by focusing only on the effect of creatinine/CysC ratio. In previous studies^5,32,33^, however, the creatinine/CysC ratio was effective as an indicator in patients with stable chronic kidney disease, implying that in the future it could be widely applied in patients other than those with acute kidney injury. Third, because of the small AUROC and low specificity, our study can only be regarded as hypothesis-generating research. Since creatinine/CysC ratio is an objective and easily measurable parameter for screening in the pre-operative period, we expect further studies with sufficient power to appropriately examine the efficacy of the creatinine/CysC ratio.

In conclusion, in this retrospective analysis, a low creatinine/CysC ratio was associated with an increased risk of developing PPCs in older patients undergoing OPCAB.

## Supplementary Information

Supplementary Tables.

## Data Availability

The datasets generated for this study are available on request to the corresponding author. The data are not publicly available due to privacy reasons.
