# Supplementary material for: Associations of creatinine/cystatin C ratio and postoperative pulmonary complications in elderly patients undergoing off-pump coronary artery bypass surgery: a retrospective study
Source: Sci Rep. 2021 Aug 19;11:16881. doi: 10.1038/s41598-021-96442-0 (PMC8376894; doi:10.1038/s41598-021-96442-0)
Supplement: Supplementary file 1 — Supplementary Tables. [file 41598_2021_96442_MOESM1_ESM.docx]

**Supplementary Materials:** The following are available online, Table S1: Comparison of preoperative laboratory data between patients with and without pulmonary complications, Table S2: Comparison of baseline characteristics and preoperative laboratory data between the high and low creatinine/cystatin C ratio groups, Table S3: Odds ratio of low creatinine/cystatin C ratio (<89.5) for the occurrence of postoperative complications, Table S4: Comparison of baseline characteristics and preoperative laboratory data between the high and low CONUT grade groups, Table S5: Odds ratio of high CONUT grade (CONUT score ≥5) for the occurrence of postoperative complications.

**Supplementary Table S1.** Comparison of preoperative laboratory data between patients with and without pulmonary complications

| **Variables** | **PPC group**  **(n=80)** | **non-PPC group**  **(n=525)** | ***P-*value** |
| --- | --- | --- | --- |
| Hemoglobin level (g/dL) | 12.4±1.6 | 12.8±1.6 | 0.051 |
| Serum albumin level (g/dL) | 3.8±0.5 | 4.0±0.4 | 0.001 |
| Serum cholesterol level (mg/dL) | 137 [115-166] | 139 [120–164] | 0.732 |
| Blood lymphocyte count (10^3^/μL) | 1.74 [1.31–2.18] | 1.79 [1.40–2.23] | 0.268 |
| Serum C-reactive protein level (mg/L) | 2.5 [0.8–6.6] | 1.7 [0.8–4.6] | 0.259 |
| Serum creatinine level (mg/dL) | 0.84±0.18 | 0.84±0.17 | 0.806 |
| Estimated GFR | 82 [71–94] | 86 [74–93] | 0.141 |

Values are presented as mean ± standard deviation or median [interquartile range]. GFR, glomerular filtration rate; PPC, postoperative pulmonary complication

**Supplementary Table S2.** Comparison of baseline characteristics and preoperative laboratory data between the high and low creatinine/cystatin C ratio groups

| **Variable** | **Grouping by creatinine/cystatin C ratio** | | ***P-*value** |
| --- | --- | --- | --- |
|  | **Low (<89.5, n=348)** | **High (≥89.5, n=257)** |  |
| **Patient** |  |  |  |
| Age (years) | 73 [68–76] | 70 [67–74] | <0.001 |
| Male sex | 194 (55.7) | 245 (95.3) | <0.001 |
| Emergency | 13 (3.7) | 2 (0.8) | 0.021 |
| BMI (kg/m^2^) | 23.9 [21.8–25.9] | 24.0 [22.3–25.6] | 0.601 |
| EuroSCORE II | 1.30 [1.04–1.72] | 1.13 [0.90–1.46] | <0.001 |
| Current smoker | 45 (12.9) | 34 (13.2) | 0.914 |
| Hypertension | 271 (77.9) | 188 (73.2) | 0.180 |
| Diabetes mellitus | 173 (49.7) | 130 (50.6) | 0.832 |
| Cerebrovascular accident | 47 (13.5) | 33 (12.8) | 0.811 |
| Congestive heart failure | 41 (11.8) | 18 (7.0) | 0.050 |
| Chronic obstructive pulmonary disease | 18 (5.2) | 12 (4.7) | 0.778 |
| Myocardial infarction within 1 month | 110 (31.6) | 72 (28.0) | 0.341 |
| Left main >50% stenosis | 91 (26.1) | 84 (32.7) | 0.080 |
| Mitral regurgitation ≥ moderate | 25 (7.2) | 7 (2.7) | 0.016 |
| Preoperative medications |  |  |  |
| Aspirin | 298 (85.6) | 222 (86.4) | 0.793 |
| Clopidogrel | 203 (58.3) | 146 (56.8) | 0.708 |
| Beta-blocker | 200 (57.5) | 136 (52.9) | 0.265 |
| Calcium channel blocker | 142 (40.8) | 115 (44.7) | 0.332 |
| RAS inhibitor | 208 (59.8) | 136 (52.9) | 0.093 |
| Insulin | 32 (9.2) | 19 (7.4) | 0.430 |
| HMG-CoA reductase inhibitors | 288 (82.8) | 195 (75.9) | 0.037 |
| Reoperation | 4 (1.1) | 1 (0.4) | 0.401 |
| Anaemia | 164 (47.1) | 104 (40.5) | 0.103 |
| Controlling nutritional status score | 2 [1-3] | 2 [1–3] | 0.717 |
| Creatinine/cystatin C ratio | 75.6 [67.9–83.1] | 100.9 [94.4–109.6] | <0.001 |
| **Preoperative Laboratory Data** |  |  |  |
| Hemoglobin level (g/dL) | 12.5±1.5 | 13.2±1.5 | <0.001 |
| Serum albumin level (g/dL) | 4.0 [3.6–4.2] | 4.1 [3.8–4.4] | <0.001 |
| Serum cholesterol level (mg/dL) | 140 [121–164] | 139 [116–165] | 0.672 |
| Blood lymphocyte count (10^3^/μL) | 1.81 [1.38–2.22] | 1.77 [1.40–2.23] | 0.724 |
| Serum C-reactive protein level (mg/L) | 2.1 [0.9–5.8] | 1.5 [0.7–4.1] | 0.013 |
| Serum creatinine level (mg/L) | 0.75 [0.64–0.88] | 0.94 [0.85–1.03] | <0.001 |
| Estimated GFR | 90 [78–96] | 80 [71–88] | <0.001 |

Values are presented as mean ± standard deviation, median [interquartile range], or the number of patients (percentages). BMI, body mass index; RAS, renin-angiotensin system; GFR, glomerular filtration rate.

| **Outcomes** | **Unadjusted OR**  **(95% CI)** | ***P*-value** | **^a^Adjusted OR**  **(95% CI)** | ***P*-value** |
| --- | --- | --- | --- | --- |
| **Pulmonary complications** | 2.88 (1.66–5.00) | <0.001 | 2.62 (1.49–4.59) | 0.001 |
| Pneumonia | 6.84 (2.05–22.84) | 0.002 | 6.48 (1.93–21.73) | 0.002 |
| Prolonged ventilation | 2.04 (1.14–3.67) | 0.017 | 1.78 (0.98–3.26) | 0.059 |
| Reintubation | 2.75 (1.01–7.51) | 0.048 | 2.44 (0.88–6.79) | 0.087 |
| **Renal failure** | 10.73 (1.40–82.14) | 0.022 | 10.36 (1.35–79.64) | 0.025 |
| **Delirium** | 1.48 (0.96–2.28) | 0.077 | 1.36 (0.88–2.11) | 0.173 |
| **Reoperation due to bleeding/tamponade** | 0.63 (0.21–1.89) | 0.406 | 0.64 (0.21–1.96) | 0.438 |
| ^b^**Permanent stroke** | Not applicable |  | Not applicable |  |
| **Mortality** | 11.53 (1.51–87.87) | 0.018 | 10.55 (1.38–80.80) | 0.023 |

**Supplementary Table S3.** Odds ratio of low creatinine/cystatin C ratio (<89.5) for the occurrence of postoperative complications

^a^ Adjusted by EuroSCORE II

^b^ Permanent stroke did not occur in the high creatinine/cystatin C ratio (≥89.5) group

OR, odds ratio; CI, confidence interval

**Supplementary Table S4.** Comparison of baseline characteristics and preoperative laboratory data between the high and low CONUT grade groups

| **Variable** | **Grouping by CONUT score** | | ***P-*value** |
| --- | --- | --- | --- |
|  | **Low (<5, n=551)** | **High (≥5, n=46)** |  |
| **Patient** |  |  |  |
| Age (years) | 72 [68–75] | 73 [68–76] | 0.132 |
| Male sex | 395 (71.7) | 37 (80.4) | 0.203 |
| Emergency | 13 (2.4) | 2 (4.3) | 0.324 |
| Body mass index (kg/m^2^) | 24.0 [22.3–25.9] | 23.3 [19.6–24.5] | <0.001 |
| EuroSCORE II, | 1.18 [0.95–1.57] | 1.56 [1.14–2.07] | 0.001 |
| Current smoker | 70 (12.7) | 7 (15.2) | 0.625 |
| Hypertension | 413 (75.0) | 39 (84.8) | 0.135 |
| Diabetes mellitus | 259 (48.8) | 28 (60.9) | 0.832 |
| Cerebrovascular accident | 69 (12.5) | 11 (23.9) | 0.029 |
| Congestive heart failure | 53 (9.6) | 6 (13.0) | 0.440 |
| Chronic obstructive pulmonary disease | 29 (5.3) | 1 (2.2) | 0.721 |
| Myocardial infarction within  1 month | 155 (28.1) | 25 (54.3) | <0.001 |
| Left main >50% stenosis | 160 (29.0) | 12 (26.1) | 0.671 |
| Mitral regurgitation ≥ moderate | 27 (4.9) | 4 (8.7) | 0.288 |
| Preoperative medications |  |  |  |
| Aspirin | 475 (86.2) | 38 (82.6) | 0.500 |
| Clopidogrel | 319 (57.9) | 27 (58.7) | 0.916 |
| Beta-blocker | 310 (56.3) | 20 (43.5) | 0.094 |
| Calcium channel blocker | 238 (43.2) | 16 (34.8) | 0.268 |
| Renin-angiotensin system inhibitor | 313 (56.8) | 26 (56.5) | 0.970 |
| Insulin | 44 (8.0) | 6 (13.0) | 0.261 |
| HMG-CoA reductase inhibitors | 434 (78.8) | 43 (93.5) | 0.017 |
| Reoperation | 5 (0.9) | 0 (0.0) | >0.999 |
| Anemia | 229 (41.6) | 33 (71.7) | <0.001 |
| Controlling nutritional status score | 2 [1-3] | 6 [5–6] | <0.001 |
| Creatinine/cystatin C ratio | 86.9 [74.3–98.1] | 82.3 [69.5–92.0] | 0.016 |
| **Preoperative Laboratory Data** |  |  |  |
| Hemoglobin level (g/dL) | 12.9 [12.0–13.9] | 11.1 [9.8–12.7] | <0.001 |
| Serum albumin level (g/dL) | 4.0 [3.8–4.3] | 3.3 [2.9–3.4] | <0.001 |
| Serum cholesterol level (mg/dL) | 142 [122–166] | 112 [95–130] | <0.001 |
| Blood lymphocyte count (10^3^/μL) | 1.86 [1.46–2.26] | 1.17 [0.90–1.36] | <0.001 |
| Serum C-reactive protein level (mg/L) | 1.7 [0.8–4.3] | 10.5 [2.2–28.6] | <0.001 |
| Serum creatinine level (mg/L) | 0.85 [0.71–0.97] | 0.81 [0.68–0.94] | 0.278 |
| Estimated glomerular filtration rate | 85 [74–93] | 89 [77–95] | 0.220 |

Values are presented as mean ± standard deviation, median [interquartile range], or the number of patients (percentages). CONUT, Controlling Nutritional Status.

**Supplementary Table S5**. Odds ratio of high CONUT grade (CONUT score ≥5) for the occurrence of postoperative complications

| **Outcomes** | **Unadjusted OR**  **(95% CI)** | ***P*-value** | **^a^Adjusted OR**  **(95% CI)** | ***P*-value** |
| --- | --- | --- | --- | --- |
| Pulmonary complications | 2.59 (1.28–5.26) | 0.008 | 2.42 (1.18–4.95) | 0.015 |
| Pneumonia | 2.93 (1.06–8.15) | 0.039 | 2.81 (1.01–7.81) | 0.048 |
| Prolonged ventilation | 2.39 (1.09–5.22) | 0.030 | 2.21 (1.00–4.91) | 0.050 |
| Reintubation | 1.15 (0.26–5.05) | 0.856 | 1.09 (0.25–4.83) | 0.913 |
| Renal failure | 0.85 (0.11–6.63) | 0.879 | 0.82 (0.11–6.38) | 0.849 |
| Delirium | 2.93 (1.54–5.54) | 0.001 | 2.77 (1.45–5.27) | 0.002 |
| Reoperation due to bleeding/tamponade | 1.00 (0.13–7.85) | 0.999 | 1.04 (0.13–8.26) | 0.971 |
| ^b^Permanent stroke | Not applicable |  | Not applicable |  |
| Mortality | 0.79 (0.10–6.15) | 0.825 | 0.76 (0.10–5.88) | 0.789 |

^a^ Adjusted by EuroSCORE II

^b^ Permanent stroke did not occur in the high CONUT grade (CONUT score ≥5)) group

CONUT, Controlling Nutritional Status; OR, odds ratio; CI, confidence interval.
